# Validation of a measure of contraceptive self-injection self-efficacy in Uganda

**DOI:** 10.1186/s12905-025-03982-y

**Published:** 2025-09-22

**Authors:** Brooke W. Bullington, Sneha Challa, Ronald Wasswa, Dinah Amongin, Catherine Birabwa, Sylvia Nanono, Peter Waiswa, Jane Cover, Kelsey Holt

**Affiliations:** 1 Division of Family Planning, Department of Obstetrics & Gynecology, University of Utah, Salt Lake City, Utah USA; 2 Institute for Health & Aging, School of Nursing, University of California San Francisco, San Francisco, CA USA; 3 School of Public Health, Makerere University, Kampala, Uganda; 4 Department of Reproductive Health, PATH, Seattle, WA USA; 5 Department of Family Community Medicine, School of Medicine, University of California San Francisco, San Francisco, CA USA

**Keywords:** Self-injectable, Self-efficacy, Self care, Contraception, Agency

## Abstract

**Background:**

Sexual and reproductive health self-care interventions, including self-administered injectable contraception, can promote individual agency and health. As an increasing number of programs seek to introduce and expand access to self-injectable contraception, it’s imperative to develop measures that define programmatic success based on whether people have the opportunity to make a decision about self-injectable use with agency, rather than relying on measures of self-injectable uptake.

**Methods:**

We performed psychometric evaluation to validate a self-injectable self-efficacy scale using cross-sectional survey data collected among 2,369 sexually active, reproductive aged women in Uganda. We examined descriptive statistics of a pool of five potential items before conducting an Exploratory Factor Analysis to determine factor structure and reduce the item pool. We ran a Confirmatory Factor Analysis to confirm the factor structure and performed a correlation analysis to determine whether the final self-injectable self-efficacy scale was correlated with items related to contraceptive decision-making self-efficacy and previous self-injectable use.

**Results:**

Our final measure of self-injectable self-efficacy is a unidimensional scale that contains three items: 1) “I feel confident that I could self-inject on my own at the end of training,” 2) “I feel confident that I can do all the steps correctly and will be protected from pregnancy,” and 3) “I can manage to self-inject even if I feel very scared at first.” Self-injectable self-efficacy was positively and significantly correlated with prior self-injectable use and items related to contraceptive decision-making self-efficacy.

**Conclusion:**

We validated a novel self-care focused measure of self-injectable self-efficacy in Uganda. Self-injectable self-efficacy, which can be measured among all reproductive-aged women regardless of prior self-injectable training or use, can be used to evaluate self-injectable programs to determine whether programs equip people with the tools needed to successfully use self-injectable contraception if they desire.

**Supplementary Information:**

The online version contains supplementary material available at 10.1186/s12905-025-03982-y.

## Background

Sexual and reproductive health (SRH) self-care practices, including those related to menstrual hygiene and fertility management, have been ongoing outside of the formal health sector for centuries [1]. The World Health Organization (WHO) defines self-care as, “the ability of individuals, families and communities to promote health, prevent disease, maintain health, and to cope with illness and disability with or without the support of a health worker” [2]. Self-care in SRH has the potential to promote individual agency, reduce health inequities, and promote overall health [3]. These interventions include self-testing for pregnancy [4], self-management of medication abortion [5–7], self-collection of samples for HIV [8, 9] or sexually transmitted infection testing [10, 11], and self-administration of injectable contraception [12], among others.

Availability of the self-injectable contraceptive method subcutaneous Depot medroxyprogesterone acetate (DMPA-SC) may reduce barriers to contraceptive access, as provider-administered injectable contraception requires a visit to a clinic or pharmacy every three months [13]. Several studies have demonstrated the efficacy and safety of self-injection (SI), finding no differences in pregnancy rates, few differences in side effects, and higher continuation rates among those using SI compared to provider-administrated injectable contraception [12, 14]. In Uganda, several studies have shown that DMPA-SC for SI is an acceptable, feasible, cost-effective form of contraceptive service delivery for women of all age groups [15–20].

Uptake of self-injectable contraception remains low across the world, even in settings like Uganda where DMPA-SC has been available for nearly a decade [21, 22]. This may be, in part, because awareness of DMPA-SC remains low in some settings or due to availability and/or affordability [23, 24]. For example, supply challenges can undermine SI programs if women are unable to obtain units to inject at home [25]. Further, research has shown that confidence to self-inject is highly dependent on the quality of SI training that providers offer, and many providers may lack the time or resources to implement high quality training [15, 26, 27].

Programs that seek to expand SI access and training have therefore been developed and implemented [28, 29]. For example, Population Services International’s Delivering Innovation in Self-Care Empathy Training extends beyond training in SI and includes community mobilization and supervision to increase user confidence [30] and Innovations for Choice and Autonomy utilizes an experienced user model to implement community-based peer support [31]. To assess whether these programs are successful in expanding access, it’s imperative to define success not in terms of uptake of SI, but by examining whether people have the opportunity to make a decision about contraception with agency [32]. Evidence from previous method introduction interventions suggests that such interventions can lead to overpromotion of new methods. For example, qualitative and quantitative studies have reported that a provider-focused postpartum intrauterine device (IUD) intervention in Tanzania led to providers overemphasizing the IUD and deemphasizing other contraceptive methods in their counseling [33, 34]. Little research has explored how introduction of DMPA-SC specifically impacts method choice. Contraceptive counseling focused on a single method and other forms of directive provider behavior may be incentivized, in part, by reliance on contraceptive uptake-focused measures and numerical targets for program evaluation. In response to this historical paradigm, scholars have repeatedly called for the development of new rights-based and person-centered measures of success for family planning programming that focus on whether people are able to fulfill their contraceptive desires [32, 35–37].

One way to assess whether programs introducing the option for SI are successful in enabling women to use this method with confidence is by measuring their self-efficacy. Wood and Bandura define self-efficacy as, “beliefs in one’s capabilities to mobilize the motivation, cognitive resources, and courses of action needed to meet given situational demands” [38]. Prior studies have developed and validated measures of contraceptive self-efficacy, which capture people’s beliefs about their capability to complete the actions needed to use contraception [39], and contraceptive decision-making self-efficacy, which focuses about self efficacy to make a decision about contraception, agnostic to what that decision is [32]. Given that use of self-administered injectable contraception requires individuals to have knowledge of proper administration techniques and confidence in their ability to self-inject, assessing whether programs can improve individuals’ self-efficacy can provide valuable insights into success of method introduction and training programs. Rather than emphasizing uptake, focusing on self-efficacy as an outcome measure in SI programming can ensure that women are provided with the information, knowledge, and support they need to self-inject successfully, should they so choose.

We therefore sought to validate a measure of contraceptive SI self-efficacy that can be used as a measure of programmatic success. We aimed to create a measure, relevant to all reproductive-aged women, that can be included in research and program evaluation. Here, we present the psychometric evaluation and validation of the contraceptive SI self-efficacy scale.

## Methods

### Item generation

The item pool for the self-injectable self-efficacy measure was informed by the General Self-efficacy Scale (GSS), which seeks to measure perceived self-efficacy in coping and adapting to daily issues [40] and has been validated in a number of African settings [41, 42], as well as by the Condom Use Self-Efficacy Scale (CUSES) [43], which seeks to measure one’s personal ability to use condoms and was developed for and applied in US settings, and later validated in Ghana [44]. We followed the general tenor of relevant items in these scales such as, “I am confident that I could deal efficiently with unexpected events” (GSS), “I feel confident that I would remember to use a condom even after I have been drinking” (CUSES-G), and “I feel confident that I could use a condom successfully” (CUSES-G), adapting items to be SI-specific. The initial item pool contained 10 items, five applicable to all reproductive-aged women and five applicable to only those who had used or taken home units of SI. Because this study sought to develop a measure relevant to all reproductive-aged women, not only those who had used or taken home units of SI, only five items were considered for the final scale.

### Study design and data collection

The item pool was tested in an observational study focused primarily in two districts in Uganda, Mayuge and Oyam. These districts were purposively selected because of the availability of DMPA-SC at public facilities and because contraceptive use was relatively low. Three additional districts, Iganga, Kole, and Lira were also included to supplement sample size, as women in these neighboring districts likely share similar characteristics to those in the primary districts. This data was collected as part of the Innovations for Choice and Autonomy project which seeks to understand women’s contraceptive preferences while optimizing facilitators and reducing barriers for accessing DMPA-SC for SI. For this measure development analysis, we used baseline survey data, which included information on contraceptive use experience, contraceptive norms, and contraceptive agency, collected from October 2022-April 2023. We recruited a purposive, convenience sample of sexually active (had sex in the past six months), reproductive-aged women (15–45 years), inclusive of contraceptive users and non-users. Currently pregnant women were not eligible for inclusion. Community health workers and health service providers were sensitized to the goals of the study and target sample: a purposive mix of contraceptive users (including SI users) and non-users. During routine operations, when they saw potentially eligible clients, they referred them to trained, female research assistants fluent in local languages to conduct a screening survey and invite them to enroll in the study. Research assistants also used client registers to identify additional eligible participants, particularly new SI users or new users of other contraceptive methods. After obtaining informed consent, research assistants conducted the survey orally with participants in a private location of the participant’s choice. Surveys took between 60 and 90 min. All data collection procedures were approved by the UCSF (#21-34470) and Makerere School of Public Health Institutional Review Boards (SPH-2022-212), and the Uganda National Council for Science and Technology (#HS2368ES).

### Measures

For the purposes of validating the self-injectable self-efficacy sale, the survey included: 1) sociodemographic characteristics, 2) the full item pool developed a priori through the item generation process, and 3) questions related to general contraceptive decision-making self-efficacy and prior SI use designated a priori to establish construct validity.

We collected participant demographic characteristics, including age, education, marital status, religion, parity, current contraceptive use, and history of self-injectable use. We measured age and parity as continuous variables, while education (none, primary, secondary, college/university), marital status (married/partnered, not currently married/partnered), religion (Catholic, Muslim, Pentecostal, Protestant, Other), current contraceptive use (currently using, currently not using), and ever use of self-injectable contraception (yes, no) were captured categorically.

We assessed a pool of five-items that were asked among all reproductive-aged women for inclusion in the self-injectable self-efficacy measure. These items were as follows: “I feel confident that I could self-inject on my own at the end of training,” “I feel confident that I can do all the steps correctly and will be protected from pregnancy,” “I can manage to self-inject even if I have to try several times until I succeed,” “I can manage to self-inject even if I do not receive a great deal of support from others,” and “I can manage to self-inject even if I feel very scared at first.” Each item had a five-point response scale: strongly agree, agree, neither agree not disagree, disagree, and strongly disagree. Items were recoded to numeric values with 4 corresponding with “strongly agree” and 0 corresponding with “strongly disagree.” Those who answered “no response” to the questions were coded as missing.

We also collected information on individual self-efficacy items that were fielded as part of the development of the contraceptive agency scale to assess convergent validity. These items were developed as part of the ICAN Measure of Contraceptive Agency [32] to capture the decision-making self-efficacy subdomain. These items include the following questions: 1) “If a family member did not support your choice, would you still be able to do what is best for you related to avoiding pregnancy?”, 2) “Are you confident that you can do what is best for you related to avoiding pregnancy?”, and 3) “If something or someone stood in your way, could you find a way to do what you want related to avoiding pregnancy?” For each of these items, participants were asked to answer “yes” or “no.”

### Psychometric evaluation

We first examined descriptive statistics, including frequencies, means, and standard deviations, for each of the five-items considered for the self-injectable self-efficacy scale. We excluded participants who were missing data for any of the five items in our initial item pool (*n* = 53, 2% of the total sample). We used an Exploratory Factor Analysis (EFA) using a random half of the full sample (EFA *n* = 1,185) to assess the factor structure of the scale. The remaining half of the sample was retained for Confirmatory Factor Analysis (CFA), which we conducted to verify the factor structure of the scale, described below.

During the EFA process, we checked the assumption of factorability using correlation matrices to ensure that all items were correlated with coefficients > 0.3. We also used the Kaiser–Meyer–Olkin test for sampling adequacy to ensure that common variance was > 0.6. We determined the factor structure and the items to retain from our pool of five items by assessing items, balancing quantitative findings with content validity. In this process, we first used Horn’s Parallel analysis to examine adjusted eigenvalues and determine the appropriate number of factors to retain. We then examined the factor structured using promax rotation, specifying the number of factors based on the results of Horn’s Parallel analysis. We removed items that did not load onto the factors with a factor loading > 0.5. During this phase, we also removed or retained items based on content validity, with the goal of retaining items that have been identified as important for self-injectable self-efficacy in the qualitative literature, including user fear of incorrectly administering SI and fear of the needle itself [15, 45, 46]. For ease of implementation, we sought to reduce the number of items for parsimony, while also maintaining coverage of the construct. We therefore aimed to reduce the item pool when possible while maintaining strong internal consistency reliability and factor loadings. We repeated these steps until we reached a factor structure that was optimized statistically and in terms of content validity. Throughout this process, we examined internal consistency reliability (measured using Cronbach’s alpha), inter-item correlations, and item-rest correlations. We sought a Cronbach’s alpha > 0.7 for the final scale. Throughout the item selection process, we examined excluded item alphas to assess if item removal impacted Cronbach’s alpha.

We used the second random half of the sample (*n* = 1,184) to verify the factor structure using CFA. We used the final set of retained items established during the EFA to fit a structural equation model using maximum likelihood estimation. We determined goal model fit indices a priori, including comparative fit index (CFI)$\ge$ 0.95, Tucker-Lewis Index (TLI)$\ge$ 0.95, root mean square error of approximation (RMSEA)$\le$ 0.6, and standardized root mean square residual (SRMR)$\le$ 0.8. We also examined suggested modification indices to assess any model changes.

To generate the final self-injectable self-efficacy scale, we took the mean of the final set of items. Individual items are represented on a scale of 0–4, with 0 indicating low self-injectable self-efficacy (i.e. respondents “strongly disagree” with the statement) and 4 indicating high self-injectable self-efficacy (i.e. respondents “strongly agree” with the statement). Using scores constructed with the final scale items, we assessed whether the final self-injectable self-efficacy model scores correlated with measures of self-injectable use and individual self-efficacy items from the contraceptive agency scale to determine validity of our measure. To do this, we regressed the mean scores of the self-injectable self-efficacy scale onto our outcomes, with robust standard errors to account for clustering by district. We present unadjusted odds ratios and 95% confidence intervals for these associations.

## Results

### Sample characteristics

This analysis included a total of *N* = 2,369 participants (Table 1). Participants had a median age of 25 years (interquartile range [IQR]: 22, 30) and a median parity of 2 (IQR: 1,4). About two-thirds of the sample (67.9%) had attended primary school and nearly all (97.5%) were currently married or partnered. About 34.2% of the sample identified as Catholic, followed by 29.6% as Protestant, 20.7% as Pentecostal, 20.7% as Muslim, and 1.6% as any other religions. Just over three quarters (78.9%) were currently using contraception, and 6.6% had ever administered the contraceptive self-injectable to themselves.

**Table 1:** Participant characteristics of reproductive-aged women, Uganda (*N* = 2,369)

	**Median**	IQR
Age (years)	25	22, 30
Parity (births)	2	1,4
	**n**	**%**
Education		
None	129	5.5%
Primary	1,609	67.9%
Secondary	533	22.5%
College/University	98	4.1%
Marital status		
Married/partnered	2,309	97.5%
Not currently married/partnered	60	2.5%
Religion		
Catholic	811	34.2%
Muslim	491	20.7%
Pentecostal	329	13.9%
Protestant	700	29.6%
Other	38	1.6%
Currently using contraception		
Yes	1,870	78.9%
No	499	21.1%
Current contraceptive method		
No method	499	21.1%
Implant	1,022	43.1%
Injectable	609	25.7%
Intrauterine device	171	7.2%
Pill	39	1.7%
Other	29	1.2%
New self-injectable user		
Yes	300	12.7%
No	2,069	87.3%
Ever self-injected contraception		
Yes	156	6.6%
No	2,213	93.4%

### Item response frequencies

The mean responses and standard deviations for all five items in the pool are shown in Table 2. Items are represented on a scale of 0–4, with 4 representing the highest contraceptive self-injectable self-efficacy and 0 representing the lowest. In our sample, item responses skew slightly toward higher self-efficacy, with all items in the initial item pool having a mean score > 2.

**Table 2:** Means and standard deviations of item pool considered for self-injectable self-efficacy scale, Uganda (*N* = 2,369)

Item number	Item	Mean	Standard Deviation
1	I feel confident that I could self-inject on my own at the end of training	2.3	1.6
2	I feel confident that I can do all the steps correctly and will be protected from pregnancy	2.6	1.4
3	I can manage to self-inject even if I have to try several times until I succeed	2.2	1.5
4	I can manage to self-inject even if I do not receive a great deal of support from others	2.2	1.5
5	I can manage to self-inject even if I feel very scared at first	2.2	1.5

### Exploratory factor analysis

Through our iterative process of assessing the scale structure and item reduction, we determined the self-injectable self-efficacy measure is unidimensional, with three items loading onto one latent factor. Two items (item 3 and item 4) were removed for parsimony and to improve content validity. We removed item 3 because it included a scenario that may not be applicable (“even if I have to try several times until I succeed”), and “try several times” is ambiguous. We removed item 4 because it also contained a scenario that may not be applicable to everyone (“even if I do not receive a great deal of support from others”) and would be difficult to answer for those who do not desire support or feel contraceptive decisions are soley theirs to make. Though item 5 contained a similar scenario that might not be applicable to all interested in SI (“even if I feel very scared at first”), we retained this item because fear was frequently mentioned in formative qualitative work. Items and factor loadings are shown in Table 3. All items loaded onto the single factor > 0.7. Cronbach’s alpha for the three-item scale was 0.92.

**Table 3:** Factor loadings from EFA and CFA for final contraceptive self-injection self-efficacy scale, Uganda (*N* = 2,369)

		**EFA**	**CFA**	
**Item number**	**Item**	**Factor loading**	**Factor loading**	**95% confidence interval**
1	I feel confident that I could self-inject on my own at the end of training	0.95	0.97	0.96, 0.98
2	I feel confident that I can do all the steps correctly and will be protected from pregnancy	0.76	0.79	0.77, 0.81
5	I can manage to self-inject even if I feel very scared at first	0.95	0.95	0.93, 0.96

### Confirmatory factor analysis

The structural equation model using the one factor extracted from the EFA showed strong support for the one-factor solution (CFI > 0.9, TLI > 0.9, RMSEA < 0.1, SRMR < 0.1) and consistently high factor loadings as observed in the EFA (Table 3). Modification indices suggested no further changes to the model specification.

### Final scale

The overall mean of the self-injectable self-efficacy scale was 2.4 (standard deviation: 1.4, range: 0–4).

### Correlation analysis

Odds ratios and 95% confidence intervals for associations between the mean self-injectable self-efficacy score and general contraceptive decision-making self-efficacy or history of self-injectable use are shown in Table 4. Mean self-injectable self-efficacy score was positively and significantly associated with a participant reporting they would still be able to do what is best for her related to avoiding pregnancy if a family member did not support her choice (OR: 1.24; 95% CI: 1.15, 1.34), feeling confident that she can do what is best for her related to avoiding pregnancy (OR: 1.35; 95% CI: 1.24, 1.46), and being able to find a way to do what she wants related to avoiding pregnancy if something or someone stood in her way (OR: 1.21; 95% CI: 1.12, 1.32). Mean self-injectable self-efficacy score was also associated with history of self-injecting (OR: 3.38; 95% CI: 2.59, 4.42).

**Table 4:** Associations between self-injectable self-efficacy score and general contraceptive decision-making self-efficacy, history of self-injectable use, Uganda (*N* = 2,369)

Outcome	Overall self-injectable self-efficacy scale	
	**Odds ratio**	**95% confidence interval**
If a family member did not support her choice, she would still be able to do what is best for her related to avoiding pregnancy	1.24	1.15, 1.34^*^
Is confident that she can do what is best for her related to avoiding pregnancy	1.35	1.24, 1.46^*^
If something or someone stood in her way, she could find a way to do what she wants related to avoiding pregnancy	1.21	1.12, 1.32^*^
Has reported ever self-administering the self-injectable	3.38	2.59, 4.42^*^

^*^Indicates *p* < 0.05

## Discussion

In the present study, we introduce the self-injectable self-efficacy scale, developed using data collected from sexually active, reproductive-aged women in Uganda. The unidimensional scale includes three items, which capture confidence to self-inject at the end of training, confidence in doing steps correctly and being protected from pregnancy, and ability to manage to self-inject even if scared at first. The measure has strong internal consistency and factor loadings for all items are high. Further, self-injectable self-efficacy is associated with higher likelihood of contraceptive decision-making self-efficacy and history of self-administering the self-injectable, providing evidence of construct validity. While other contraception-related scales measure self-efficacy [32, 39], the self-injectable self-efficacy scale is novel in that it specifically focuses on self-care.

The self-injectable self-efficacy scale provides an alternative outcome measure for evaluating programs that seek to introduce or train people to use the self-administered contraceptive injectable. While initiatives to increase contraceptive access have historically evaluated programs using indicators of method uptake like increased contraceptive prevalence or decreased unmet need, scholars have emphasized the need for more person-centered, rights-based outcomes that capture ability to fulfill contraceptive desires, agency, and autonomy [32, 35, 36, 47]. This is especially important in the context of introducing new contraceptive methods, given that the goal of new method introduction is to expand method choice rather than increase the prevalence of any one given method. Our measure of self-injectable self-efficacy offers program implementers and evaluators the ability to capture whether participants feel confident and capable of self-administering the injection, regardless of whether they ultimately decide to use self-injectable contraception, another form of contraception, or no contraceptive method at all. SI self-efficacy relies on and trusts participants’ perception of their SI knowledge and confidence, thereby centering the individual and their views in the measurement approach. Because the scale can be administered to all reproductive-aged women, regardless of history of self-injectable training or use, the measure can be captured both before and after introduction of or training on self-injectable, allowing program evaluators to examine differences in self-injectable self-efficacy pre- and post-intervention. SI self-efficacy was not designed to specifically reflect desire to use SI, but like other measures of general contraceptive self-efficacy, it likely correlates with the extent to which a person may want to use contraception or SI. Those who implement the measure should consider that levels of SI self-efficacy may never reach 100%.

This study used a large, diverse sample of reproductive aged women in Uganda, including contraceptive users and non-users, to develop and evaluate self-injectable self-efficacy. It also has several limitations. While items on the self-injectable self-efficacy score were developed based on other general self-efficacy scales and literature on women’s specific fears about injecting themselves, there was limited opportunity for cognitive testing prior to survey implementation. Thus, while all items included a “neither agree nor disagree” response option for those who do not feel equipped to answer, there is an opportunity for future work to assess the comprehension and interpretation of items. This testing should account for women’s contraceptive use and SI of DMPA-SC use experiences. Additionally, while the item pool used for scale construction was relatively small, the psychometric properties of SI self-efficacy are strong, suggesting that this limitation did not affect our ability to identify well-performing items. Further, our use of a convenience sample limits the generalizability of the findings within Uganda, and the lack of data from other countries limits our ability to assess the degree to which it is valid for use in other settings.

## Conclusions

In conclusion, we developed the self-injectable self-efficacy scale, a valid and reliable measure of self-efficacy in self-administering the contraceptive injectable among reproductive-aged women in Uganda. This is a rights-based scale that offers an alternative measure of success for programs that seek to introduce or train individuals on the self-administered injectable. Self-injectable self-efficacy is an innovative self-care focused measure that captures whether people are able to use self-injectable contraception, thereby expanding their contraceptive method choice.

## Supplementary Information

Supplementary Material 1: Supplementary Table 1. Characteristics of reproductive-aged women in EFAand CFA samples, Uganda. Supplementary Table 2. Mean responses and standard deviations for survey items among all reproductive-aged participants and those who had ever used or taken home units of self-injectable contraception.

## Data Availability

The dataset used and/or analyzed during the current study are available from the corresponding author on reasonable request.

## Abbreviations

CI: Confidence Interval
CFA: Confirmatory Factor Analysis
CFI: Comparative fit index
CUSES: Condom Use Self-Efficacy Scale
DMPA-SC: Depot Medroxyprogesterone Acetate-Subcutaneous
EFA: Exploratory Factor Analysis
GSS: General Self-efficacy Scale
IQR: Interquartile Range
OR: Odds Ratio
RMSEA: Root Mean Square Error of Approximation
SI: Self-Injectable
SRH: Sexual and Reproductive Health
SRMR: Standardized Root Mean Square Residual
TLI: Tucker-Lewis Index
WHO: World Health Organization
