# Supplementary material for: Validation of a measure of contraceptive self-injection self-efficacy in Uganda
Source: BMC Womens Health. 2025 Sep 22;25(Suppl 1):431. doi: 10.1186/s12905-025-03982-y (PMC12452008; doi:10.1186/s12905-025-03982-y)
Supplement: Supplementary file 1 — Supplementary Material 1: Supplementary Table 1. Characteristics of reproductive-aged women in EFAand CFA samples, Uganda. Supplementary Table 2. Mean responses and standard deviations for survey items among all reproductive-aged participants and those who had ever used or taken home units of self-injectable contraception. [file 12905_2025_3982_MOESM1_ESM.docx]

**Supplementary Table 1.** Characteristics of reproductive-aged women in EFA (n=1,185) and CFA samples (n=1,184), Uganda

|  |  | |  |  |
| --- | --- | --- | --- | --- |
|  |  | |  |  |
|  | **EFA sample** | | **CFA sample** | |
|  | **Median** | **IQR** | **Median** | **IQR** |
| **Age** (years) | 25 | [21, 30] | 25 | [22, 31] |
| **Parity** (births) | 2 | [1, 4] | 2 | [1, 4] |
|  | **n** | **%** | **n** | **%** |
| **Education** |  |  |  |  |
| None | 66 | 5.6% | 63 | 5.3% |
| Primary | 797 | 67.3% | 812 | 68.3% |
| Secondary | 276 | 23.3% | 257 | 21.7% |
| College/University | 46 | 3.9% | 52 | 4.4% |
| **Marital status** |  |  |  |  |
| Married/partnered | 1,164 | 98.2% | 1,145 | 96.7% |
| Not currently married/partnered | 21 | 1.8% | 39 | 3.3% |
| **Religion** |  |  |  |  |
| Catholic | 412 | 34.8% | 399 | 33.7% |
| Muslim | 253 | 21.4% | 238 | 20.1% |
| Pentecostal | 173 | 14.6% | 156 | 13.2% |
| Protestant | 329 | 27.8% | 371 | 31.3% |
| Other | 18 | 1.5% | 20 | 1.7% |
| **Currently using contraception** |  |  |  |  |
| Yes | 934 | 78.8% | 936 | 79.1% |
| No | 251 | 21.2% | 248 | 21.0% |
| **Current contraceptive method** |  |  |  |  |
| No method | 251 | 21.2% | 248 | 21.0% |
| Implant | 509 | 43.0% | 513 | 43.3% |
| Injectable | 310 | 26.2% | 299 | 25.3% |
| Intrauterine device | 84 | 7.1% | 87 | 7.4% |
| Pill | 17 | 1.4% | 22 | 1.9% |
| External condom | 1 | 0.1% | 2 | 0.2% |
| Standard Days Method | 0 | 0.0% | 2 | 0.2% |
| Lactational Amenorrhea Method | 2 | 0.2% | 0 | 0.0% |
| Rhythm method | 0 | 0.0% | 1 | 0.1% |
| Emergency contraception | 0 | 0.0% | 1 | 0.1% |
| Female sterilization | 1 | 0.1% | 0 | 0.0% |
| Male sterilization | 0 | 0.0% | 0 | 0.0% |
| Internal condom | 0 | 0.0% | 0 | 0.0% |
| Withdrawal | 0 | 0.0% | 0 | 0.0% |
| Diaphragm | 0 | 0.0% | 0 | 0.0% |
| Foam/Jelly | 0 | 0.0% | 0 | 0.0% |
| Other | 10 | 0.8% | 10 | 0.8% |
| **Ever self-injected contraception** |  |  |  |  |
| Yes | 84 | 7.1% | 72 | 6.1% |
| No | 1,101 | 92.9% | 1,112 | 93.9% |

|  |  | **All Women of Reproductive Aged** | | **Participants who had ever used or taken home units of SI** | |
| --- | --- | --- | --- | --- | --- |
| **Item number** | **Item** | **Mean** | **Standard Deviation** | **Mean** | **Standard Deviation** |
| 1 | I feel confident that I could self-inject on my own at the end of training | 2.3 | 1.6 | 3.5 | 0.99 |
| 2 | I feel confident that I can do all the steps correctly and will be protected from pregnancy | 2.6 | 1.4 | 3.6 | 0.74 |
| 3 | I can manage to self-inject even if I have to try several times until I succeed | 2.2 | 1.5 | 3.4 | 0.98 |
| 4 | I can manage to self-inject even if I do not receive a great deal of support from others | 2.2 | 1.5 | 3.4 | 0.97 |
| 5 | I can manage to self-inject even if I feel very scared at first | 2.2 | 1.5 | 3.4 | 0.95 |
| 6 | I feel confident that I know how to activate the device | - | - | 3.5 | 0.94 |
| 7 | I feel confident that I know how to create a skin tent at the injection site | - | - | 3.5 | 0.99 |
| 8 | I feel confident that I can inject the contraceptive into my body | - | - | 3.5 | 0.99 |
| 9 | I feel confident in pressing the reservoir to expel the liquid | - | - | 3.5 | 0.93 |
| 10 | I feel confident in calculating my re-injection date | - | - | 3.5 | 0.92 |

**Supplementary Table 2.** Mean responses and standard deviations for survey items among all reproductive-aged participants and those who had ever used or taken home units of self-injectable contraception.
